# Leber's hereditary optic neuropathy – case report and literature review

**DOI:** 10.1590/S1516-31802004000600010

**Published:** 2004-11-04

**Authors:** Hélio Afonso Ghizoni Teive, André Ribeiro Troiano, Salmo Raskin, Lineu César Werneck

**Keywords:** Optic neuropathy, Optic nerve diseases, Optic atrophy, Mitochondrial DNA, Visual acuity, Neuropatia óptica, Doenças do nervo óptico, Atrofia óptica, DNA mitocondrial, Acuidade visual

## Abstract

**CONTEXT:**

Leber's hereditary optic neuropathy is an important cause of progressive painless visual loss among young male patients.

**OBJECTIVE:**

To report on a case of a young patient with a clinical and neurophysiological condition suggestive of Leber's hereditary optic neuropathy, confirmed by genetic testing.

**CASE REPORT:**

We describe a 17-year-old male with progressive bilateral visual loss. Two maternal uncles had had similar patterns of visual loss. The patient had a history of smoking and alcohol abuse. Neuro-ophthalmological examination revealed visual acuity of 20/800 in both eyes, with decreased direct and consensual pupillary light reflexes. Fundus examination demonstrated pale optic discs. The visual evoked potential test showed signs of conduction disturbances in both optic nerves and campimetric study showed complete visual loss in all fields of both eyes. A diagnosis of bilateral optic neuropathy with a clinical suspicion of Leber's hereditary optic neuropathy was made. A blood sample was submitted to genetic analysis in relation to the principal mutations of this disorder, and homoplasmic mutation in 11778 was detected, thereby confirming the diagnosis of Leber's hereditary optic neuropathy.

## RESUMO

**CONTEXTO:**

A neuropatia óptica hereditária de Leber representa uma importante causa de perda visual progressiva, sem dor, em pacientes jovens do sexo masculino.

**OBJETIVO:**

Relatar o caso de um paciente jovem com quadro clínico e neurofisiológico sugestivo de neuropatia óptica hereditária de Leber, confirmado pelo teste genético.

**RELATO DE CASO:**

Jovem do sexo masculino com 17 anos de idade com perda visual bilateral progressiva tinha história familiar de perda visual progressiva em dois tios maternos. O paciente tinha antecedentes de tabagismo e alcoolismo pesado. O exame neuro-oftalmológico demonstrou acuidade visual de 20/800 em ambos os olhos, com diminuição dos reflexos pupilares direto e consensual e palidez de papilas ópticas no exame de fundo de olho. O exame de potencial visual evocado definiu distúrbio de condução em ambos os nervos ópticos. O exame de campimetria visual revelou perda visual completa em todos os campos visuais de ambos os olhos. O paciente foi diagnosticado com neuropatia óptica bilateral, com a suspeita clínica de neuropatia óptica hereditária de Leber, e o estudo genético para a avaliação das principais mutações encontradas nesta doença revelou a presença da mutação homoplásmica 11778, confirmando, desta forma, o diagnóstico de neuropatia óptica hereditária de Leber.

## INTRODUCTION

Leber's hereditary optic neuropathy is a neuro-ophthalmological entity characterized by acute or subacute bilateral visual loss that is usually sequential, with centrocecal scotoma and occasional further visual improvement. It is associated with a matrilineal inheritance pattern. Its diagnosis used to be solely clinical, aided by imaging and neurophysiological studies, until the advent of descriptions of mitochondrial biochemical abnormalities and genetic testing.^1-3^ We present the case of a young male whose symptoms were compatible with Leber's hereditary optic neuropathy, as confirmed by genetic testing.

## CASE REPORT

A 17-year-old male was brought to our medical attention complaining of visual loss. Six months earlier, he had first lost sight in his left eye, over the course of a few days, until he could see only shadows. One week later, this symptom occurred in his right eye. At the time of our initial consultation, he said that he was only able to see shadows in his right eye.

This patient had had generalized tonic-clonic seizures from the age of seven months until he was two years old, with good control using phenobarbital. When he was a child, he had mumps and chickenpox. Two maternal uncles had had similar patterns of visual loss, but they died of non-related diseases, without a definite diagnosis, when they were 50 years old. The patient had been smoking about 30 cigarettes/day and drinking heavily every day for one year, but had quit these habits when the visual symptoms began.

Neuro-ophthalmological examination revealed visual acuity of 20/800 in both eyes. The direct and consensual pupillary light reflexes were decreased, whereas the extrinsic ocular motility was normal. A campimetric study showed complete visual loss in all fields of both eyes. Fundus examination demonstrated pale optic discs.

The patient was initially given the diagnosis of bilateral optic neuritis and, over the last six months, he has been submitted to the following tests in the ophthalmology unit: (1) visual campimetry, which showed marked decrease of retina sensitivity in both eyes; (2) magnetic resonance imaging of the skull and orbits, which was normal; (3) cerebrospinal fluid examination, which showed normal cells absence, glucose of 70 mg/dl, proteins of 178 mg/dl, normal protein electrophoresis and also the oligoclonal banding; (4) visual evoked potential (during the first days of the disease), which showed discrete signs of conduction disturbances in the optic nerves; (5) retina angiofluorescein, which showed signs of pale optic discs bilaterally, associated with tortuous retinal vessels. The patient underwent pulse therapy using methylprednisolone 1 g/day for three days, and maintenance therapy of prednisone 40 mg/day, without any visual improvement.

When he was admitted into our unit, we decided to perform muscle biopsy (left brachial biceps muscle), with the intention of searching for abnormalities suggestive of mitochondrial cytopathy, such as the presence of ragged-red fibers. The results were abnormal but nonspecific, with atrophy of fiber types 1 and 2. Our clinical suspicion of Leber's hereditary optic neuropathy remained high and a blood sample was therefore submitted to genetic analysis in relation to the principal mutations of this disorder.

After DNA extraction from peripheral blood cells, using traditional methods, we proceeded with molecular analysis to investigate four primary points of mitochondrial DNA (11778, 3460, 14484 and 15257). These were analyzed using the polymerase chain reaction (PCR), restriction enzyme digestion of DNA (restriction fragment length polymorphism) and the direct sequencing method, under the conditions depicted in Table 1. Homoplasmic mutation in 11778 was detected, thereby confirming our diagnostic hypothesis.

**Table 1:** Testing conditions for polymerase chain reaction for the detection of mitochondrial DNA mutations in a case of Leber's hereditary optic neuropathy

Mutation	Method	Fragment	Hybridization	Enzyme	Incubation	Detection
G11778A	RFLP	11325 — 12209 (1008)	56° C	Mae III	1H - 55° C	Acrylamide 8%
G3460A	RFLP	3201 — 3600 (399)	62° C	Bsa HI	1-2H — 37° C	Acrylamide 8%
G14484A	RFLP	14401 — 14510 (109)	56° C	Bsr I	1H - 65° C	Acrylamide 11%
	Sequencing	13901 — 14976 (1075)	58° C	primer sequencing		
T15257	RFLP	14401 — 15927 (1526)	58° C	Acc I	1-2H — 37° C	Agarose 1%

*RFLP = Restriction fragment length polymorphism.*

During outpatient follow-up, his symptoms have not increased or decreased, and he has been medicated using vitamin C (500 mg t.i.d.), vitamin B_1_ (5 mg), vitamin B_2_ (2 mg), vitamin B_6_ (2 mg), vitamin PP (20 mg), vitamin B_5_ (3 mg), in one tablet, t.i.d, and coenzyme Q10 (100 mg b.i.d.). The purpose of this supplementation was to improve cellular ATP usage.

## DISCUSSION

Hereditary optic neuropathies are a group of diseases with defined clinical presentation and different inheritance patterns. The clinical setting may be of acute, sub-acute or relentlessly progressive painless visual loss, bilateral (simultaneous or sequential), with centrocecal scotoma, altered color perception (dyschromatopsia) and optic atrophy. The inheritance pattern may present as autosomally dominant, recessive, X-linked or matrilineal.^1^

In Leber's hereditary optic neuropathy, male individuals in their teens or twenties suffer acute visual loss that is sequential in 78% of cases and simultaneous in 22%.^2^ Fundus examination in the initial stages shows papilledema and peripapillary microangiopathy, evolving to atrophy of the nerve fiber layer of the retina and finally leading to optic atrophy and centrocecal scotoma.^3^ Family history is suggestive of maternal inheritance in 50% of patients, and in the other 50% the disease seems to be sporadic.^2,4^

Four main mutations of mitochondrial DNA (mtDNA) encompass over 90% of patients with Leber's hereditary optic neuropathy: 11778 (genetic subunit ND4), 14484 (ND6), 3460 (ND1) and 14459 (ND6). The mutation at 14459 corresponds to the dystonia phenotype for Leber's hereditary optic neuropathy.^2,5^ Mashima et al.^6^ assessed the prevalence of different mutations in a sample of 80 individuals with Leber's hereditary optic neuropathy and showed that 87% carried the mutation at 11778, 9% at 14484 and 4% at 3460. Riordan-Eva et al.^2^ found prevalences for the same mutations of respectively 75%, 15% and 8%. Oriental studies demonstrate higher proportions of 11778 mutations than do Western studies.^6,7^ Biousse et al.^8^ reported the 14484 mutation on monozygotic twins with distinct phenotypes (only one symptomatic sibling), while genetic testing in the mother was negative, thereby suggesting de *novo* mutation.

Sadun et al. recently published an extensive investigation of a large Brazilian lineage of 11778/haplogroup J with Leber's hereditary optic neuropathy. The authors emphasized the strong influence of environmental risk factors, with smoking as the most common factor.^9^

Eighty-five percent of patients with Leber's hereditary optic neuropathy are homoplasmic for mtDNA mutations; the remaining 15% are heteroplasmic. The relationship between heteroplasmy levels and clinical condition was studied by Chinnery et al.,^10^ who showed that heteroplasmic patients with higher degrees of mutant mtDNA had a greater probability of developing visual loss. Women carrying up to 80% mutant mtDNA are less prone to have symptomatic sons than are homoplasmic women. Nevertheless, this has been refuted by Huoponen,^3^ on the basis that heteroplasmy levels probably differ between nerve cells and white blood cells. Moreover, environmental influences also contribute to the optic nerve damage.

Secondary mutations such as 3394, 4216, 4917, 5244, 7444, 9438, 13708 and 15257 have been studied with regard to the genesis of Leber's hereditary optic neuropathy. Recent studies have not found any difference in the prevalence of secondary mutations between individuals with Leber's hereditary optic neuropathy and the control group.^6,7^

Multiple sclerosis (or MS-like disease) shares a rather uncommon comorbidity with Leber's hereditary optic neuropathy. Some patients with Leber's hereditary optic neuropathy develop clinical features that are phenotypically indistinguishable from multiple sclerosis, and mutations for Leber's neuropathy are considered to be a risk factor in the pathophysiology of multiple sclerosis.^11^

On the other hand, prevalence studies among multiple sclerosis patients have failed to demonstrate primary mtDNA mutations.^12,13^ It is currently recommended to proceed with mtDNA analysis for all young male multiple sclerosis patients with initial neuro-ophthalmological manifestations and peripapillary microangiopathy, especially those with bilateral symptoms and a positive family history for visual loss.^14^

There is no specific treatment for Leber's hereditary optic neuropathy. From an empirical standpoint, most if not all patients will receive an initial diagnosis of optic neuritis and will be treated, without any response, using steroid therapy at high doses. In a case-control study with patients carrying mutations 14484, 3460 and 11778, combination therapy using idebenone/vitamin B_2_/vitamin C (which improves ATP availability) shortened the time required for vision recovery in the group using this therapy (11.1 months), in comparison with the placebo group (17.4 months; p = 0.03).^15^ A single-patient study backed by magnetic resonance spectroscopy of the central nervous system (31P-MRS) showed clinical and imaging improvement when idebenone treatment was instituted (idebenone is not available in Brazil, but only in Argentina: its action is similar to that of the coenzyme Q10).^16^

The prognosis for vision recovery depends on the mutation reported. Good prognosis may be found in up to 50% of patients bearing the 14484 mutation. Nevertheless, only 4% of patients with the 11778 mutation have gradual improvement, as we were able to observe in our patient.^2,17^

## CONCLUSION

A diagnosis of Leber's hereditary optic neuropathy should be suspected whenever young males develop bilateral visual loss, usually sequential, with a positive familial history. Neuro-ophthalmological examination may demonstrate suggestive signs of Leber's hereditary optic neuropathy. The mutation at the 11778 locus is the most frequent mutation of mitochondrial DNA in Leber's hereditary optic neuropathy patients.

## References

[1] Kerrison JB (2001). Hereditary optic neuropathies. Ophthalmol Clin North Am.

[2] Riordan-Eva P, Sanders MD, Govan GG, Sweeney MD, Da Costa J, Harding AE (1995). The clinical features of Leber's hereditary optic neuropathy defined by the presence of a pathogenic mitochondrial DNA mutation. Brain.

[3] Huoponen K. (2001). Leber hereditary optic neuropathy: clinical and molecular genetic findings. Neurogenetics.

[4] Yamada K, Mashima Y, Kigasawa K, Miyashita K, Wakakura M, Oguchi Y (1997). High incidence of visual recovery among four Japanese patients with Leber's hereditary optic neuropathy with the 14484 mutation. J Neuroophthalmol.

[5] Brown MD, Allen JC, Van Stavern GP, Newman NJ, Wallace DC (2001). Clinical, genetic, and biochemical characterization of a Leber hereditary optic neuropathy family containing both the 11778 and 14484 primary mutations. Am J Med Genet.

[6] Mashima Y, Yamada K, Wakakura M (1998). Spectrum of pathogenic mitochondrial DNA mutations and clinical features in Japanese families with Leber's hereditary optic neuropathy. Curr Eye Res.

[7] Yen MY, Wang AG, Chang WL, Hsu WM, Liu JH, Wei YH (2002). Leber's hereditary optic neuropathy – the spectrum of mitochondrial DNA mutations in Chinese patients. Jpn J Ophthalmol.

[8] Biousse V, Brown MD, Newman NJ (1997). De novo 14484 mitochondrial DNA mutation in monozygotic twins discordant for Leber's hereditary optic neuropathy. Neurology.

[9] Sadun AA, Carelli V, Salomão SR (2003). Extensive investigation of a large Brazilian pedigree of 11778/haplogroup J Leber hereditary optic neuropathy. Am J Ophthalmol.

[10] Chinnery PF, Andrews RM, Turnbull DM, Howell NN (2001). Leber hereditary optic neuropathy: Does heteroplasmy influence the inheritance and expression of the G11778A mitochondrial DNA mutation?. Am J Med Genet.

[11] Vanopdenbosch L, Dubois B, D'Hooghe MB, Meire F, Carton H (2000). Mitochondrial mutations of Leber's hereditary optic neuropathy: a risk factor for multiple sclerosis. J Neurol.

[12] Pénisson-Besnier I, Moreau C, Jacques C, Roger JC, Dubas F, Reynier P (2001). Sclérose en plaques et mutations de l'ADN mitochondrial associées à la maladie de Leber. [Multiple sclerosis and Leber's hereditary optic neuropathy mitochondrial DNA mutations]. Rev Neurol (Paris).

[13] Kalman B, Lublin FD, Alder H (1995). Mitochondrial DNA mutations in multiple sclerosis. Mult Scler.

[14] Mojon DS, Herbert J, Sadiq SA, Miller JR, Madonna M, Hirano M (1999). Leber's hereditary optic neuropathy mitochondrial DNA mutations at nucleotides 11778 and 3460 in multiple sclerosis. Ophthalmologica.

[15] Mashima Y, Kigasawa K, Wakakura M, Oguchi Y (2000). Do idebenone and vitamin therapy shorten the time to achieve visual recovery in Leber hereditary optic neuropathy?. J Neuroophthalmol.

[16] Cortelli P, Montagna P, Pierangeli G (1997). Clinical and brain bioenergetics improvement with idebenone in a patient with Leber's hereditary optic neuropathy: a clinical and 31P-MRS study. J Neurol Sci.

[17] Nakamura M, Yamamoto M (2000). Variable pattern of visual recovery of Leber's hereditary optic neuropathy. Br J Ophthalmol.

